# Vitamin B_6_ Status Assessed by Plasma Vitamers and Homocysteine in Lactating Dairy Cows and Its Relationship to Rumen Fermentation, Plasma Metabolites, and Milk Production in Different Environmental Conditions

**DOI:** 10.1111/asj.70084

**Published:** 2025-07-22

**Authors:** Suttida Prombood, Taketo Obitsu, R‐Jun Frederick Avelino Gaspe, Toshihisa Sugino, Yuzou Kurokawa, Thanutchaporn Kumrungsee

**Affiliations:** ^1^ Graduate School of Integrated Sciences for Life Hiroshima University Higashi‐Hiroshima Japan; ^2^ Agriculture Department Capiz State University Pilar, Capiz Philippines

**Keywords:** automatic milking system, homocysteine, hot environment, pyridoxal phosphate, pyridoxic acid

## Abstract

The supply of vitamin B_6_ from diet and rumen microbial synthesis is generally considered to be sufficient in dairy cows. However, increased milk yield and environmental factors may alter their vitamin B_6_ status. This study aimed to clarify the vitamin B_6_ status in lactating dairy cows milked with automatic milking system (AMS) under different environmental conditions. In the winter and summer feeding experiments, plasma concentrations of vitamin B_6_ vitamers of pyridoxal (PL) and pyridoxal‐5′‐phosphate (PLP) with pyridoxic acid (PA), and homocysteine (Hcys) of the cows were assessed, along with their feed intake, milk production, rumen fermentation, and plasma metabolites. Higher PL but lower PA concentrations and PA/(PLP + PL) ratio in plasma were found in the summer experiment compared with the winter experiment, even though plasma PLP concentration, milk production, and dry matter intake were similar between the winter and summer experiments. Plasma concentration of vitamin B_6_ vitamers and Hcys concentration did not correlate with milk production parameters. However, the PA/(PLP + PL) ratio in plasma negatively correlated with ruminal acetic acid composition. Summer environment and the variation of rumen fermentation could alter the turnover of vitamin B_6_, even though plasma PLP concentration is likely maintained in lactating cows milked with AMS.

## 1 Introduction

Vitamin B_6_ is a water‐soluble vitamin and an essential micronutrient required for maintaining physiological functions and production in animals. Vitamin B_6_ plays a crucial role in amino acid metabolism as a coenzyme of various reactions including transamination and transsulfuration pathways and is vital for protein synthesis. Although symptoms of vitamin B_6_ deficiency are rarely observed in mature ruminants, calves fed a diet low in vitamin B_6_ exhibit signs of reduced appetite, anorexia, impaired growth and, in severe cases, epileptiform seizures and mortality (Johnson et al. 1950). In animals, vitamin B_6_ exists in several interconvertible vitamer forms, including pyridoxine (PN), pyridoxal (PL), and pyridoxamine (PM), along with their phosphorylated derivatives, such as pyridoxal‐5′‐phosphate (PLP, the active form) and its catabolic byproduct, pyridoxic acid (PA) (Parra et al. 2018).

In lactating dairy cows, vitamin B_6_ supply is generally considered to adequately meet the requirements through combination supplies of dietary intake and microbial synthesis in the rumen (NRC 2001). However, the amount of vitamin B_6_ entering the small intestine is lower than that ingested due to the ruminal degradation and the negative apparent ruminal synthesis, which is affected by dietary composition and dietary vitamin B_6_ intake (Castagnino et al. 2016; Seck et al. 2017).

Vitamin B_6_ acts as a coenzyme in the conversion of homocysteine (Hcys) into cysteine (Cys), which is a precursor for the synthesis of antioxidant substance glutathione (Hsu et al. 2015). A study has shown that cattle exposed to heat stress reduce their whole‐blood glutathione levels (Lakritz et al. 2002). Additionally, Hcys is also re‐methylated to produce methionine (Met), a possible limiting amino acid for milk protein synthesis and milk production in dairy cows (Patton 2010). During heat stress, amino acid utilization, which relates to vitamin B_6_, increases to support glucose metabolism in dairy cows (Gao et al. 2017). Thus, we hypothesized that elevated environmental temperature during summer may potentially have an impact on the vitamin B_6_ status of dairy cows. Furthermore, advancements in genetic selection and the adoption of advanced management technologies, such as automatic milking systems (AMS), have significantly increased milk yield over the past decade (Rotz et al. 2003). These several factors may increase vitamin B_6_ requirement of dairy cows as suggested by Robinson (2019).

However, determining vitamin B_6_ status in dairy cows remains complex due to the various factors affecting the balance between the supply through dietary intake, ruminal degradation, microbial synthesis and absorption, and the demands for nutrient metabolism in the tissues. Generally, vitamin B_6_ status has been assessed based on direct and indirect functional biomarkers (Ueland et al. 2015). Plasma concentrations of vitamin B_6_, particularly PLP concentration, can be used as a direct biomarker of vitamin B_6_ status. However, relying solely on PLP may not provide a complete evaluation, as other phosphorylated and nonphosphorylated vitamers contribute to the systemic homeostasis (Footitt et al. 2013). It has been suggested that additional indirect functional biomarkers should be incorporated to more accurately assess vitamin B_6_ status (Ueland et al. 2015). Plasma Hcys can be used as an indirect biomarker of vitamin B_6_ status, as its concentration is reported to elevate with vitamin B_6_ deficiency (Ubbink et al. 1996). Therefore, this study aims to clarify the vitamin B_6_ status of dairy cows milked with AMS under different environmental conditions. In this study, plasma concentrations of vitamin B_6_ vitamers of PL and PLP with PA, and Hcys of the cows were assessed and compared between the winter and summer experiments, along with their milk production, rumen fermentation and plasma metabolites to evaluate environmental impact on vitamin B_6_ status in dairy cows. In addition, the relationships between these markers and parameters were analyzed to clarify the factors affecting vitamin B_6_ status in lactating dairy cows.

## 2 Materials and Methods

### 2.1 Experimental Condition

All experiment procedures complied with the guidelines established by the Animal Care and Use Committee of Hiroshima University and received official approval (No. 21‐1). Two feeding experiments were conducted in Hiroshima University Experimental Farm (Higashihiroshima, Japan) during different seasons in late November 2021 (winter experiment) and late August 2022 (summer experiment). In both experiments, cows were housed in a free‐stall barn equipped with an automatic feeder with door feeders for individual cows (RIC system, HokoFarm Group, Emmelord, the Netherlands), AMS, and large ventilation fans. The AMS was renewed after the winter experiment in March 2022. Cows were allowed to access freely to the AMS for milking and their own door feeder.

In both experiments, approximately 30 lactating cows were raised in the barn, and cows were fed partial mixed rations (PMR), with slightly different ingredient compositions between two experiments (Table 1). Specifically, dehydrated corn silage was included in the ration used in the summer experiment. The PMR was formulated to meet total digestible nutrients (TDN) requirements of cows, with 69% TDN content (dry matter [DM] basis) both in the winter and summer experiments, following the feeding standard (NARO 2017). The PMR was delivered via an automatic feeder into the feed trough of the door feeder, maintaining a 5% refusal. In addition to PMR, cows received a concentrate diet (Table 1) dispensed through a feeder of AMS during milking. The concentrate diet was provided from 3 to 8 kg/day, as a fed basis, depending on individual milk yield or days after parturition. Cows were provided free access to water and mineral blocks.

**TABLE 1:** Ingredients and nutrient contents of partial mixed ration (PMR) and concentrate diet provided at milking for cows used in the winter and summer experiments.^a^

Item	PMR			
	Winter	Summer	Concentrate diet	
Ingredient, % of DM				
Italian ryegrass silage	31.8	22.1		
Dehydrated corn silage	—	11.5		
Oat hay	10.2	9.9		
Alfalfa hay	13.2	12.8		
Formula feed	43.2	41.6		
Calcium carbonate	0.5	0.5		
Vitamin supplements (A, D, and E)	0.7	0.7		
Sodium bicarbonate supplements	0.1	0.5		
Salt	0.3	0.3		
Nutrient content				
DM, % of fresh matter	70.5	62.8	89.9	
Crude protein, % of DM	13.3	13.3	17.3	
aNDF_OM_, % of DM	47.2	46.6	20.5	
Ether extract, % of DM	3.44	4.30	2.70	
Estimated TDN, % of DM	69.0	69.0	85.7	

Abbreviations: aNDF_OM_, ash free neutral detergent fiber; DM, dry matter; TDN, total digestible nutrients.

^a^ The winter and summer experiments were conducted during late November and late August, respectively.

In the winter experiment, a total of 29 Holstein lactating cows (179 ± 104.0 days in milk (DIM, average ± SD); 18 primiparous and 11 multiparous) were selected from the lactating cow group for the assessment during 1 week of the measurement period after 2 weeks of the adjustment period. The PMR was provided by splitting four equal portions at 1000 h, 1200 h, 1400 h and 1600 h. Cows were milked by the Lely AMS (Astronaut A3 plus, Lely, Drachten, the Netherlands). In the AMS, cows received concentrate diet only when milking was allowed.

In the summer experiment, a total of 18 Holstein lactating cows (184 ± 107.8 DIM (average ± SD); 3 primiparous and 15 multiparous) were selected for the assessment during 1 week of the measurement period after 2 weeks of the adjustment period. The supply of PMR was distributed in three portions daily at 1000, 1300, and 1600 h with a ratio of 20:35:45 as a fed basis. Cows were assigned to feed‐weigh trough as similar to winter experiment and were milked using the renewed AMS (VMS V300, DeLaval, Tumba, Sweden). In the AMS used in the summer experiment, the concentrate diet was offered whenever cows entered the AMS, regardless of milking permission: cows entering AMS without milking permission were allowed to stay for 2 min with receiving small portion of concentrate diet. The setting of milking permission interval at AMS was almost same between the winter and summer experiments.

Barn temperature (T) and relative humidity (RH) were measured throughout the experimental period with a logger (GL240, Graphtec Corporation, Yokohama, Japan) attached with a thermocouple sensor and humidity sensor. The logger and sensors were placed beside the door feeders in the barn where the wind from fans was not affected. The T and RH were recorded every 10‐min intervals and the daily mean values were calculated. The temperature‐humidity index (THI) was calculated using the following equation (NRC 1971): THI = (1.8 × T + 32) − (0.55 − 0.0055 × RH) × (1.8 × T − 26), where T is in degrees Celsius (°C) and RH is in percentage (%), and daily mean THI was calculated as well.

### 2.2 Sampling and Data Collection

The PMR intake was monitored using individual feed troughs (RIC system), while concentrate diet intake at AMS was recorded automatically via AMS. The PMR samples were collected daily at the first feed delivery to determine DM content, while concentrate diet samples were collected once during the measurement period. All samples were dried at 70°C in an air‐forced oven to determine DM content. Daily DM intake of PMR and concentrate were calculated using recorded daily feed intake and the DM content of each sample. Daily milk yield and milking frequency were recorded via AMS, and milk samples were collected at each milking for 24 h on a day during the measurement period. In the winter experiment, body weight was automatically recorded by AMS during every milking. In the summer experiment, it was measured once by a weighing scale placed beside the barn when blood samples were collected. The daily feed intakes for the PMR and concentrate, the daily milk yields, and the body weights (only for the winter experiment) of individual cows were averaged over 7 days of the measurement period.

Blood samples were collected from the tail artery using a vacutainer between 1300 h and 1400 h on a day during the measurement period. Samples were centrifuged at 2330× *g* for 15 min at 4°C, and the supernatant plasma was stored at −80°C for later analysis of plasma vitamin B_6_ vitamers, Hcys, Cys, Met, and biochemical parameters.

The rumen fluid was collected using a stomach tube at the same time of blood collection. In the winter experiment, 17 of 29 cows were selected (*n* = 17; parity, 1.5 ± 0.9; milk yield 43.6 ± 8.0), while all cows (*n* = 18; parity, 2.0 ± 0.6; milk yield 41.1 ± 8.8) were used for rumen fluid sampling in the summer experiment. Within 30 min after collection, the rumen fluid was filtered through a four‐layer gauze. The filtrate was stored at −30°C for volatile fatty acids (VFA) analysis.

### 2.3 Chemical Analysis

#### 2.3.1 Feed and Milk Composition

Dried PMR and concentrate samples were ground in a Wiley mill through a 1‐mm sieve. Samples were then analyzed for DM, crude protein (CP), crude ash, and ether extract content according to AOAC (1999). Ash‐free neutral detergent fiber (aNDF_OM_) was determined using the fiber bag system (Fiberthem, Gerhardt, Königswinter, Germany). Milk samples were analyzed for milk fat, protein, and lactose contents using an infrared analyzer (Lactoscope Filter C4+, Delta Instruments, Drachten, the Netherlands).

#### 2.3.2 Plasma Vitamin $B_{6}$

Vitamin $B_{6}$ concentrations in plasma were analyzed for PLP, PN, PM, PL, and PA with a modified fluorometric method described by Tsuge (1997) and Kumrungsee et al. (2019), using HPLC system (Jasco, Tokyo, Japan) with a fluorescence detector (FP‐4025, Jasco). For standard solutions, PLP (P9255, Sigma‐Aldrich, St. Louis, MO, USA), PN (P5669, Sigma‐Aldrich), PM (P9380, Sigma‐Aldrich), PL (hydrochloride, P9130, Sigma‐Aldrich), and PA (P‐9620, Sigma‐Aldrich) were dissolved in ultrapure water and diluted to obtain mixed standard solutions. Plasma samples were deproteinized with 1 mol/L cold perchloric acid in a 2:1 ratio, vortexed for 30 s, and centrifuged at 12,000× *g* at 4°C for 10 min. The corrected supernatant was mixed with 0.2 mol/L sodium‐phosphate buffer, and pH was adjusted to 7.5 with potassium hydroxide solution before a second centrifugation at 12,000× *g* at 4°C for 5 min. The supernatant was split into two parts: one for measuring PN, PM, PL, and PA concentrations using a fluorometric detection system with emission and excitation wavelengths of 390 nm and 350 nm, respectively. The other part was used for PLP analysis after converting PLP to pyridoxic‐5′‐phosphate by treatment with 0.1 mol/L potassium cyanide, incubation at 50°C for 3 h, and storage at 4°C for 24 h. The PLP derivatives was quantified by the emission and excitation wavelengths at 420 nm and 320 nm, respectively. Samples of the first part and second derivatives were separately injected into the HPLC system equipped with a Cosmosil 5 $C_{18}$‐MS‐II column (4.6 ID × 150 mm) with the same condition: an isocratic elution of 1% (v/v) acetonitrile‐0.1 mol/L NaClO_4_–0.1 mol/L phosphate buffer (pH 3.5) at the constant flow rate of 1 mL/min. This procedure succeeded to identify and quantify PLP, PN, PM, PL, and PA in standard solution. However, for plasma samples, the concentration of PN and PM could not be determined, due to their low concentrations or the appearance of interference peaks around the retention time of PN and PM on the chromatograph. Therefore, the concentrations of PLP, PL, and PA in plasma were shown in the result table. Similarly, PL and PA could not be determined in some cases by our HPLC system (see Table 4).

#### 2.3.3 Plasma Homocysteine, Cysteine, and Methionine

Plasma concentrations of Hcys, Cys, and Met were measured according to the isotope dilution method described by Ding et al. (2021) and MacCoss et al. (1999). Briefly, after spiking with 50 μL of an internal standard solution of 10 μM [H28]‐homocystine (3,3,3′,3′,4,4,4′,4′‐$d_{8}$, 98%, DLM‐3619‐1; Cambridge Isotope Laboratories Inc., Tewksbury, MA, USA), 100 μM [H24]‐cystine (3,3,3′,3′,‐$d_{4}$, 98%, DLM‐1000‐1; Cambridge Isotope Laboratories Inc.), and 100 μM [H23]‐methionine (methyl‐$d_{3}$, 98%, DLM‐431‐1; Cambridge Isotope Laboratories Inc.) into 200 μL of plasma, dithiothreitol was added to reduce all disulfide bonds of homocystine and cystine. Subsequently, samples were deproteinized with an acetic acid solution (1 mol/L) and obtained supernatant was desalted with AG50W X8 (Bio‐Rad). The purified free amino acids were derivatized by adding 80 μL of acetonitrile/MTBSTFA (*N*‐*tert*‐butyldimethylsilyl‐*N*‐methyl‐trifluoroamide, > 97%, 394,882‐25ML; Sigma‐Aldrich; 1:1, v/v). The derivatized amino acids were measured with a gas chromatograph‐mass spectrometer (GC/MS‐QP2010; Shimadzu, Kyoto, Japan) attached with DB‐5 ms column (25 m length, 2.5 mm ID) via electron ionization at 70 eV. The column temperature program started at 160°C for 5 min, increased to 280°C at 15°C/min, then to 300°C, at 30°C/min. The fragment ions were measured by selected‐ion monitoring for unlabeled homocysteine (*m/z* = 420), cysteine (*m/z* = 406), methionine (*m/z* = 320), and the internal standard of reduced [H24]‐homocysteine (*m/z* = 424), [H22]‐cysteine (*m/z* = 408), and [H23]‐methionine (*m/z* = 323). The concentrations of these amino acids were calculated with standard curves between area ratios and concentrate ratios of labeled and unlabeled amino acids.

#### 2.3.4 Plasma Biochemical Profile and Ruminal Volatile Fatty Acids

Plasma biochemical profile (triglyceride [TG], total cholesterol, nonesterified fatty acids [NEFA], urea nitrogen [BUN], and glucose) was analyzed using an automated biochemical analyzer (AU480 Backman Coulter, CA, USA). Ruminal VFA concentrations were measured using a gas chromatograph (GC 2014, Shimadzu, Kyoto, Japan). For deproteinization, 1 mL of ruminal fluid was mixed with 0.2 mL of 20% (w/w) metaphosphoric acid, then centrifuged at 10,000× *g* for 10 min at 4°C. The supernatant was spiked with 4‐methyl valeric acid as an internal standard and analyzed with a BP21 column (25 m length, 0.53 mm ID, 0.5 μm thickness, Trajan Scientific and Medical, Victoria 3134, Australia) with following conditions: injector temperature 280°C, detector temperature 280°C, gas pressure 60 kPa. The column temperature program started at 80°C for 2 min, increased to 120°C at 10°C/min, then to 124°C, at 1°C/min, and finally to 230°C at 30°C/min.

### 2.4 Statistical Analysis

All statistical analyses were conducted using JMP 18 Pro (SAS Institute Inc., Cary, NC, USA). From the winter and summer experiments, 47 observations in total were obtained for most of parameters except for the VFA concentration (*n* = 35 in total) due to the limited number of sampling cows. For intake data (*n* = 46 in total), data from one cow were missed due to problem in recording. For plasma vitamin B_6_ concentration, one cow was not detected for plasma PLP (*n* = 46 in total). The number of observations for PL and PA was less than 47 because four samples were undetectable due to low concentrations that could not be detected by our HPLC system. For remaining 43 samples, some values were unusually high or low; thus, the outlier analysis was applied, and seven values were excluded based on *Z*‐scores greater than |±3| with careful consideration.

To evaluate the baseline levels of plasma vitamin B_6_ in lactating dairy cows, pooled data from both winter and summer experiments were subjected to distribution analysis to assess variability. Subsequently, the average, maximum, minimum values, and standard deviation for each parameter along with other observed parameters were calculated. The variation in parameters was also calculated as the coefficient of variation (CV), which was calculated by dividing the standard deviation (SD) by the mean and expressed as a percentage (%CV = [SD/mean] × 100).

To evaluate the impacts of environmental condition including raising management factors, statistical differences in the simple averages of variables between winter and summer experiments were examined using *t*‐tests. In addition, correlation analysis was performed to evaluate relationships between plasma vitamin B_6_ and feed intake, milk production, rumen fermentation, and plasma variables using pooled data obtained from the winter and summer experiments. Prior to this, correlation analyses were performed separately for each experiment (winter and summer) to identify the relationship trends within each dataset. This step was taken to confirm that the trends were similar across experiments before pooling the data. For the *t*‐test and correlation analysis, statistical significance was set at *p* < 0.05.

In this study, several factors, such as the type and management of feeds, lactation stage and parity of cows, and the type of AMS, were confounded with thermal conditions in the winter and summer experiments. Therefore, pooled data were subjected to principal component analysis (PCA) to find the relationship between plasma vitamin B_6_ markers and the created principal components (PC) representing variation of parameters. Observed parameters with factor loadings of |≥ 0.50| were considered to have a strong association to each PC.

## 3 Results and Discussion

### 3.1 Environmental Conditions

In this study, the data were obtained from lactating cows in a cold environment of the winter season and in a hot environment of the summer season. During the measurement period of the winter experiment, the averages of daily mean T, daily mean RH, and daily mean THI were 7.7°C, 62.4%, and 48.3, respectively. These conditions were not uncomfortable cold environments for cows because cold stress conditions are considered to occur when THI is less than 25 (Xu et al. 2015). In contrast, during the measurement period of the summer experiment, the average of these parameters reached 27.5°C, 79.1%, and 78.6, respectively, suggesting that cows experienced heat stress during this period, because the THI value higher than 72 regards as heat stress condition of dairy cows (Armstrong 1994).

### 3.2 Feed Intake and Milk Production and Composition

In both experiments, the daily amount of concentrate diet at AMS was provided based on individual milk yield or days after parturition. The PMR was also provided individually based on TDN requirement determined by milk yield, milk composition, and body weight. However, the average intake of concentrate diet was numerically higher (*p* = 0.12) in the winter experiment (6.2 ± 1.0 kg/day DM) compared with the summer experiment (5.7 ± 1.2 kg/day DM, Table 2). Conversely, mean PMR intake was numerically lower (*p* = 0.12) in the winter (19.6 ± 2.7 kg/day DM) than in the summer experiment (21.5 ± 4.2 kg/day DM), even though there were no statistical differences in both the concentrate and PMR intake between the two experiments. The trend in PMR intake may have reflected a compensatory intake pattern, where cows consuming less concentrate diet may increase their intake of PMR. In addition, the difference in time and frequency of PMR delivery may have also influenced PMR intake between the winter and summer experiments. However, the clear reasons for these differences remain unclear, especially considering the variation in dietary composition between two experiments.

**TABLE 2:** Means, variation, and comparison of obtained data in the winter and summer experiments for parity, days in milk, body weight, feed intake, and milk production of cows.^a^

Item	Pooled data					Experiment comparison^b^		
	*n*	Mean	SD	Min	Max	Winter	Summer	*p*
Parity	47	1.8	0.8	1.0	4.0	1.6 ± 0.9	2.0 ± 0.6	0.09
Days in milk, day	47	181	105	23	413	179 ± 104	184 ± 108	0.89
Body weight, kg	47	667	73.5	545	908	677 ± 77	650 ± 65	0.20
Intake, kg/day								
Dry matter intake								
PMR	46	20.3	3.4	14.2	30.5	19.6 ± 2.7	21.5 ± 4.2	0.12
Concentrate	47	6.0	1.1	3.4	7.8	6.2 ± 1.0	5.7 ± 1.2	0.12
Total DMI	46	26.3	3.8	17.6	37.3	25.8 ± 3.3	27.1 ± 4.6	0.32
Crude protein intake	46	3.7	0.5	2.5	5.2	3.7 ± 0.5	3.8 ± 0.6	0.36
TDN intake	46	19.2	2.8	12.8	26.9	18.9 ± 2.4	19.7 ± 3.3	0.39
Milk production, kg/day								
Milk yield	47	40.6	8.8	18.2	57.5	40.3 ± 9.0	41.1 ± 8.8	0.77
ECM yield	47	39.0	7.9	17.6	56.5	38.7 ± 8.3	39.5 ± 7.3	0.71
Milk component yield, kg/day								
Fat	47	1.5	0.4	0.4	2.4	1.4 ± 0.4	1.5 ± 0.4	0.34
Protein	47	1.4	0.4	0.6	1.9	1.4 ± 0.3	1.3 ± 0.2	0.14
Lactose	47	1.9	0.4	0.9	2.7	1.9 ± 0.4	1.9 ± 0.4	0.62
Milk component, %								
Fat	47	3.6	0.7	1.5	5.4	3.5 ± 0.6	3.8 ± 0.8	0.28
Protein	47	3.4	0.4	2.8	4.2	3.6 ± 0.3	3.3 ± 0.4	0.01
Lactose	47	4.7	0.2	4.3	5.1	4.8 ± 0.1	4.6 ± 0.1	< 0.01
Milking frequency	47	2.7	0.5	1.7	4.0	2.6 ± 0.4	2.9 ± 0.7	0.07

Abbreviations: DMI, dry matter intake; ECM, energy corrected milk; Max, maximum; Min, minimum; *n*, number of observation; PMR, partial mixed diet; SD, standard deviation; TDN, total digestible nutrients.

^a^ The winter and summer experiments were conducted during late November and late August, respectively.

^b^ Means of the winter and summer experiments were compared by *t*‐test.

There were individual variations of milk yield (Table 2) in the winter (milk yield range; 18.2–57.5 kg/day, CV = 22.3%) and summer experiments (milk yield range; 24.8–55.9 kg/day, CV = 21.4%), due to the variation of DIM and parities of cows used in both experiments (Table 2). Despite the numerical differences in mean DMI of concentrate diet and PMR, the mean of milk yield did not differ between the winter (40.3 ± 9.0 kg/day) and summer experiments (41.1 ± 8.8 kg/day, Table 2). However, the contents of milk protein and milk lactose were significantly lower (*p* ≤ 0.01) in the summer experiment compared with the winter experiment (Table 2). The mean of milking frequency was numerically greater (*p* = 0.07) in the summer experiment compared to the winter experiment (Table 2). In the summer experiment, the concentrate diet was offered whenever cows entered the AMS even milking was not permitted. This may have encouraged cows to visit the AMS, potentially contributing to the increased milking frequency observed in the summer experiment. Higher milking frequency is associated with enhancing daily milk yield and milk component yield (Astuti et al. 2015). Furthermore, the effective cooling was provided by large fans in the barn during the summer experiment. Additionally, the higher proportion of multiparous cows in the summer (multiparous: primiparous ratio = 5.0) compared to the winter experiment (the ratio = 0.6). Previous study has indicated that milk production increases with parity (Lee and Kim 2006). Overall, these factors may have contributed to the similar lactation performance except for milk protein and lactose content between the two experiments, even though cows suffered under heat‐stressed condition in the summer experiment.

### 3.3 Ruminal Volatile Fatty Acids and Plasma Biochemical Profile

In ruminants, the primary source of energy comes from VFA produced by the microbial fermentation of ingested feed in the rumen (Bergman 1990). A high‐fiber diet primarily promotes the acetic acid production, whereas a high‐grain diet increases the propionic acid level in the rumen. Additionally, a diet rich in starch enhances the butyric acid formation. In this study, the total VFA concentration and the proportion of acetic acid were greater (*p* < 0.01) for cows in the summer experiment than those in the winter experiment, but the proportion of butyrate showed an opposite trend (Table 3). The rumen fermentation patterns and microbial activity are known to be affected by the heat stress (Yadav et al. 2013). Although difference in feed intake between the two experiments were not statistically significant, cows in the summer experiment showed numerically higher PMR intake, along with slightly lower concentrate intake as explained above (Table 2). These dietary differences, combined with elevated ambient temperatures and humidity, may have shifted rumen fermentation toward a more fiber‐based fermentation profile, favoring increased acetate and reduced butyrate production. In addition, the lower rumen passage rate or prolonged fermentation under heat stress may cause to increase in total VFA concentration in the summer experiment (Yadav et al. 2013).

**TABLE 3:** Means, variation, and comparison of obtained data in the winter and summer experiment for ruminal volatile fatty acid profiles of cows.^a^

Item	Pooled data					Experiment comparison^b^		
	*n*	Mean	SD	Min	Max	Winter	Summer	*p*
Total VFA, mmol/L	35	112.2	16.3	75.3	135.8	101.0 ± 14.2	122.8 ± 9.9	< 0.01
VFA proportion, mol/100 mol								
Acetic acid	35	63.4	2.3	59.4	69.6	62.2 ± 1.5	64.5 ± 2.4	< 0.01
Propionic acid	35	21.8	3.1	16.6	27.4	21.4 ± 2.7	22.1 ± 3.5	0.53
Butyric acid	35	11.6	2.2	7.8	15.9	13.1 ± 1.6	10.3 ± 1.7	< 0.01

Abbreviations: Max, maximum; Min, minimum; *n*, number of observation; SD, standard deviation; VFA, volatile fatty acids.

^a^ The winter and summer experiments were conducted during late November and late August, respectively.

^b^ Means of the winter and summer experiments were compared by *t*‐test.

The plasma concentrations of biochemical components were shown in Table 4. The CV observed in TG (36.6%), total cholesterol (19.8%), NEFA (66.5%), and ketone bodies (23.6%) may reflect the wide ranges of the lactation stage of cows as well as a difference in the experimental condition between the two experiments. These plasma biochemical concentrations align with values previously reported for lactating dairy cows (Ardalan et al. 2011; Joo et al. 2021). When the mean values of the two experiments were compared, the plasma concentration of TG, urea nitrogen and ketone bodies were significantly lower (*p* < 0.01) for the summer experiment than those for the winter experiment. The lower concentration of plasma ketone bodies observed may be partly associated with a reduced ruminal proportion of butyric acid in summer (Table 3), as a precursor for ketone body synthesis.

**TABLE 4:** Means, variation and comparison of obtained data in the winter and summer experiments for plasma concentrations of vitamin B_6_ vitamers and metabolites of cows.^a^

Item	Pooled data					Experiment comparison^b^		
	*n*	Mean	SD	Min	Max	Winter	Summer	*p*
Plasma vitamin B_6_, nmol/L								
Pyridoxal‐5′‐phosphate (PLP)	46	362	134.2	179	706	372 ± 113 (29)^c^	345 ± 167 (17)	0.55
Pyridoxal (PL)	36	117	61.6	32	304	86 ± 36 (20)	155 ± 65 (16)	< 0.01
Pyridoxic acid (PA)	36	281	239.4	48	801	394 ± 232 (23)	81 ± 31 (13)	< 0.01
PA/(PLP + PL) ratio	29	0.60	0.5	0.10	1.81	0.83 ± 0.4 (19)	0.17 ± 0 (10)	< 0.01
Plasma metabolites								
Homocysteine, μmol/L	47	6.0	1.4	3.8	10.6	5.6 ± 1.0	6.7 ± 1.7	0.03
Cysteine, μmol/L	47	99.1	10.6	74.5	120.6	95 ± 8.8	106 ± 9.8	< 0.01
Methionine, μmol/L	47	23.7	4.5	11.7	32.3	25 ± 4.8	22 ± 4.1	0.10
Triglyceride, mg/dL	47	6.9	2.5	2.0	15.9	7.9 ± 2.6	5.3 ± 1.3	< 0.01
Total Cholesterol, mg/dL	47	242.9	48.2	125.3	365.7	251.7 ± 49.7	228.5 ± 43.3	0.05
NEFA, μmol/L	47	158.3	105.2	69.4	678.4	148.8 ± 82.6	173.7 ± 135.4	0.49
Urea nitrogen, mg/dL	47	13.4	2.1	8.8	17.8	14.3 ± 1.8	12.1 ± 1.8	< 0.01
Glucose, mg/dL	47	65.1	5.1	54.0	75.5	66.2 ± 4.6	63.2 ± 5.4	0.05
Ketone bodies, μmol/L	47	585.2	138.0	305.0	957.5	634.8 ± 122.9	505.4 ± 125.4	< 0.01

Abbreviations: NEFA, nonesterified fatty acids; *n*, number of observation; SD, standard deviation; Min, minimum; Max, maximum.

^a^ The winter and summer experiments were conducted during late November and late August, respectively.

^b^ Means of the winter and summer experiments were compared by t‐test.

^c^ Figures in parentheses indicate the number of effective observation excluding undetected measurement and outlier values for each vitamer.

### 3.4 Plasma Concentration of Vitamin B_6_ and Pyridoxic Acid

The vitamin B_6_ status in animals could be affected by the level of vitamin B_6_ intake. However, dietary B_6_ intake was not measured in this study, because estimating the actual intestinal supply of vitamin B_6_ is particularly difficult in ruminants. This is due to the extensive ruminal degradation of dietary vitamin B_6_ (Castagnino et al. 2016; Seck et al. 2017) and interconversion of the vitamers in the rumen (Santschi, Chiquette, et al. 2005), making it difficult to accurately assess the effective intestinal supply of vitamin B_6_ based on the information of dietary vitamin B_6_ intake.

In ruminants, PN and PL are provided from diets as well as microbial synthesis in the rumen (Santschi, Chiquette, et al. 2005). Even though PN is the predominant form of vitamin B_6_ in diets, PL and PM account for large proportions of vitamin B_6_ flowing to the duodenum of cows (Santschi, Berthiaume, et al. 2005). In the intestinal tissue in mice, absorbed PN and PM are converted to PL which is released to bloodstream (Sakurai et al. 1991). Therefore, plasma PL can be considered as an indicator of vitamin B_6_ status in dairy cows. In this study, pooled means of the plasma concentration of PL (117 nmol/L, Table 4) were higher than those previously reported in calves (7.8–96 nmol/L), goat (65 nmol/L), and sheep (57 nmol/L) (Coburn et al. 1984; Dubeski et al. 1996; Dubeski and Owens 1993).

Plasma PLP concentration is considered to reflect the tissue concentration of PLP which is formed from PL by the phosphorylation enzyme or from the phosphorylated forms of PN and PM by the oxidase enzyme in the tissues (Parra et al. 2018). In ruminants, plasma PLP concentrations have been reported across species, ranging from 57 to 402 nmol/L in calves, 651 nmol/L in goats, and 986 nmol/L in sheep (Coburn et al. 1984; Dubeski et al. 1996; Dubeski and Owens 1993). In mid‐lactation dairy cows, the average plasma vitamin B_6_ concentration is 53 nmol/L (Sato et al. 2003). In this study, as shown in Table 4, plasma PLP concentrations (179–706 nmol/L) were likely within the range reported previously in ruminants.

Pyridoxic acid is derived from the catabolism of PL and is considered the final oxidation product of vitamin B_6_, primarily excreted in the urine (Yagi et al. 2010). Pyridoxic acid is often used as an indicator of vitamin B_6_ intake and overall vitamin B_6_ status (Hansen et al. 1996). In this study, plasma PA concentrations (48–801 nmol/L, Table 4) were comparable with those previously reported in calves (23–91 nmol/L), sheep (318 nmol/L), and goats (105 nmol/L) (Coburn et al. 1984; Dubeski et al. 1996; Dubeski and Owens 1993). The plasma PA/(PLP + PL) ratio is recognized as a marker of vitamin B_6_ turnover, indicating the rate at which vitamin B_6_ is metabolized, utilized, and excreted (Ueland et al. 2015). In this study, as presented in Table 4, the PA/(PLP + PL) ratio ranged from 0.10 to 1.81, with a mean of 0.60. The variation (CV = 78.3%) reflected the variability in plasma concentrations of PLP, PL, and PA.

In this study, differences in plasma vitamin B_6_ concentrations were identified between the two experiments, with significantly higher plasma PL and lower PA concentrations observed in the summer experiment compared to the winter experiment (*p* < 0.01). Despite elevated ambient temperature or different raising conditions in the summer experiment, plasma PLP concentrations remained comparable between the two experiments. Although the mechanism underlying the stable plasma PLP concentration and increasing plasma PL in the summer experiment is not fully understood, the following possible explanations are proposed. First, elevated plasma PL may serve as a reservoir to support PLP synthesis under heat stress. Previous research has reported that summer heat stress enhances amino acid metabolism to support glucose utilization in dairy cows (Gao et al. 2017). In humans, higher PLP concentrations have been associated with lower plasma glucose levels (Hou et al. 2011), suggesting that increased plasma PL levels may represent a compensatory response to elevated metabolic demands for PLP, especially in relation to amino acid and glucose metabolism during heat stress. Second, the elevated plasma PL in the summer experiment may be attributed to a reduced conversion of PL to PA, indicating a conservation mechanism facilitate preserving PL for PLP synthesis. This hypothesis is supported by the concurrent decrease in plasma PA concentrations in the summer experiment. Because PA is the oxidized form of PL, a significant negative correlation (*r* = −0.42, *p* < 0.05) was observed between the plasma concentration of PA and that of PL (Table 5), which indicates the increasing PL concentration by reducing the oxidation to PA.

**TABLE 5:** Correlation coefficients among plasma concentrations of vitamin B_6_ vitamers.

Item	PL	PA	PA/(PLP + PL) ratio
Pyridoxal‐5′‐phosphate (PLP)	0.02	0.29	0.21
Pyridoxal (PL)		−0.42*	−0.52*
Pyridoxic acid (PA)			0.94**

* *p* < 0.05.

** *p* < 0.01.

Furthermore, the PA/(PLP + PL) ratio was significantly lower in the summer experiment than in the winter experiment (*p* < 0.01), suggesting a decrease in vitamin B_6_ turnover in the summer. This may reflect a physiological adaptation in vitamin B_6_ metabolism specifically in its utilization, conversion, or degradation rate as a strategy to conserve vitamin B_6_ supply for metabolic reaction response to a hot environment as described above. However, the influence of dietary composition or other factors on the difference in plasma B_6_ concentrations between the two experiments should not be excluded.

### 3.5 Plasma Concentration of Homocysteine, Cysteine, and Methionine

Because Hcys is converted to Cys via PLP‐dependent enzymes, PLP insufficiency potentially elevates plasma Hcys and reduces Cys concentrations. Methionine serves as a key methyl donor, and it is essential for milk protein synthesis (Wei et al. 2024). In addition, Hcys is also used for Met resynthesis by accepting the methyl group from the folic acid cycle. Therefore, plasma concentrations of Hcys, Cys, and Met have a strong relationship with each other.

In a previous report, serum Hcys concentrations in various cattle breeds were reported to range from 16.4 to 17.4 μmol/L (Kiliçkap and Kozat 2017). Additionally, dairy cows with low ruminal pH (pH < 5.5) were reported to have an average plasma Hcys concentration of 3.9 μmol/L (Cannizzo et al. 2012). Preynat et al. (2009) reported mean plasma concentrations of Hcys, Cys, and Met as 5.1, 105, and 21 μmol/L, respectively, in lactating Holstein cows producing an average milk yield of 38 kg/day. Duplessis et al. (2022) reported plasma concentrations of Hcys, Cys, and Met as 3.5, 86, and 27 μmol/L, respectively, in postpartum dairy cows with an average milk yield of 40 kg/day. Although physiological differences may influence plasma concentration of Hcys, Cys, and Met, the concentrations observed in this study (Table 4) fall within the ranges reported in the previous studies.

In this study, significantly higher (*p* < 0.05) plasma concentrations of Hcys and Cys, along with a numerically lower (*p* = 0.10) concentration of Met, were observed in the summer experiment compared to the winter experiment (Table 4). The elevation for the plasma concentrations of both Hcys and Cys and reducing trend of plasma Met concentration, despite stable plasma PLP concentrations, indicate the reduction of remethylation of Hcys to Met, associated with limiting supply of methyl group (e.g., insufficiency of vitamin B_12_ or folate, Selhub et al. 2007). As a result, less regeneration of Met from Hcys possibly contributes to the lower milk protein composition observed in the summer experiment (Table 2), given that Met is a key limiting amino acid for milk protein synthesis.

Simultaneously, the increase in plasma Cys in the summer experiment may reflect enhanced transulfuration of Hcys to Cys via a PLP‐dependent mechanism. The consistent plasma PLP concentration between the two studies indicates sufficient coenzyme availability to support this reaction, maintaining the synthesis of Cys, a precursor for glutathione, which plays a critical role in cellular antioxidant defense. This suggests a potential metabolic adaptation during summer, whereby the pathway shifts from methionine synthesis toward transsulfuration to enhance antioxidant capacity in response to oxidative stress associated with high ambient temperatures.

### 3.6 Relationship of Plasma Vitamin B_6_ Markers With Milk Production and Nutrient Metabolism

To investigate the association between vitamin B_6_ status and parameters related to milk production and nutrient metabolism, correlation analyses with the pooled data were performed. Plasma concentrations of Hcys and vitamin B_6_ vitamers, including PA, did not show any significant correlations with total DMI and milk yield (Table 6), even though plasma Hcys showed significant negative correlation (*p* < 0.05) with concentrate intake and yields of milk protein and lactose. These results imply that vitamin B_6_ seems to be sufficient to cover the increased milk production, because presumably higher demands of vitamin B_6_ with increasing milk production did not cause the increase of plasma Hcys concentration or reduction of plasma concentrations of vitamin B_6_ vitamers, which are potential symptoms of vitamin B_6_ insufficiency (Ueland et al. 2015).

**TABLE 6:** Correlation coefficients of plasma concentrations of vitamin B_6_ vitamers and homocysteine with dry matter intake, milk production, plasma metabolite concentrations, and rumen fermentation parameters.

Item	Pyridoxal‐5′‐phosphate (PLP)	Pyridoxal (PL)	Pyridoxic acid (PA)	PA/(PLP + PL)	Homocysteine
Days in milk	−0.06	0.03	−0.03	−0.06	0.15
Body weight	0.19	−0.23	0.13	0.11	0.24
Dry matter intake					
PMR	0.00	0.07	−0.16	−0.15	−0.12
Concentrate	0.15	−0.07	0.23	0.18	−0.32*
Total DMI	0.04	0.04	−0.07	−0.07	−0.20
Crude protein intake	0.05	0.03	−0.06	−0.06	−0.06
TDN intake	0.05	0.04	−0.04	−0.06	−0.22
Milk production					
Milk yield	0.06	0.06	0.06	−0.06	−0.26
Milk component yield					
Fat	0.10	0.02	0.04	0.01	−0.13
Protein	0.08	−0.06	0.22	0.03	−0.32*
Lactose	0.08	−0.05	0.12	0.03	−0.31*
ECM yield	0.10	0.02	0.08	−0.01	−0.22
Milking frequency	0.07	0.14	−0.03	−0.06	−0.22
Plasma metabolite concentration					
Homocysteine	0.10	0.24	−0.33	−0.41*	—
Cysteine	−0.06	0.24	−0.60*	−0.58*	0.60*
Methionine	0.06	0.07	0.11	0.12	0.24
Triglyceride	0.07	−0.17	0.39*	0.37	−0.07
Total cholesterol	−0.06	0.06	0.34*	0.27	−0.18
NEFA	−0.06	−0.14	−0.09	−0.06	−0.05
Urea nitrogen	0.15	−0.08	0.37*	0.23	−0.15
Glucose	−0.04	−0.32	0.05	0.20	0.27
Ketone bodies	0.21	−0.32	0.30	0.30	0.03
Total VFA concentration	−0.07	0.35	−0.27	−0.36	0.24
VFA proportion					
Acetic acid	0.11	0.07	−0.42*	−0.49*	0.50*
Propionic acid	−0.11	0.27	0.05	−0.05	−0.25
Butyric acid	0.03	−0.43*	0.36	0.51*	−0.13

Abbreviations: DMI, dry matter intake; ECM, energy corrected milk; NEFA, nonesterified fatty acids; PMR, partial mixed ration; TDN, total digestible nutrients; VFA, volatile fatty acids.

* *p* < 0.05.

However, significant correlations were observed between the plasma vitamin B_6_ markers and various plasma metabolites and rumen VFA. Plasma PL showed a significant negative correlation with the ruminal proportion of butyric acid (*r* = −0.43, *p* < 0.05), suggesting that the increased plasma PL observed during the summer experiment may be partially attributed by a reduced production of butyrate in the rumen. Plasma PA exhibited a significant negative correlation with the proportion of ruminal acetic acid (*r* = −0.42, *p* < 0.05), while plasma Hcys showed significant positive correlation with ruminal acetic acid (*r* = 0.50, *p* < 0.05). Consequently, the PA/(PLP + PL) ratio showed significant negative correlation with the ruminal proportion of acetic acid (*r* = −0.49, *p* < 0.05) and positively correlated with the proportion of butyric acid (*r* = 0.51, *p* < 0.05). These relationships indicate that shift to the fiber‐based fermentation profile observed in the summer experiment as described above would result in reducing vitamin B_6_ catabolism, which is possibly another reason for elevated plasma PL concentration in the summer experiment.

In contrast, plasma PA showed significant positive correlations with plasma TG, total cholesterol, and blood urea nitrogen (*r* = 0.39, 0.34, and 0.37, respectively, *p* < 0.05), highlighting a potential role for vitamin B_6_ in the lipid and protein metabolism. Therefore, the observed reductions in plasma TG, total cholesterol, and blood urea nitrogen in the summer experiment may partially associate with lower vitamin B_6_ degradation.

Furthermore, plasma PA/(PLP + PL) showed significant negative correlation with plasma Hcys (*r* = −0.41, *p* < 0.05) and Cys (*r* = −0.58, *p* < 0.05), suggesting that suppression of vitamin B_6_ turnover resulted in elevated concentrations of Hcys and Cys. This may reflect a physiological mechanism to preserve PLP for use in the transsulfuration reactions to form Cys, under metabolic stress during summer.

**FIGURE 1:**
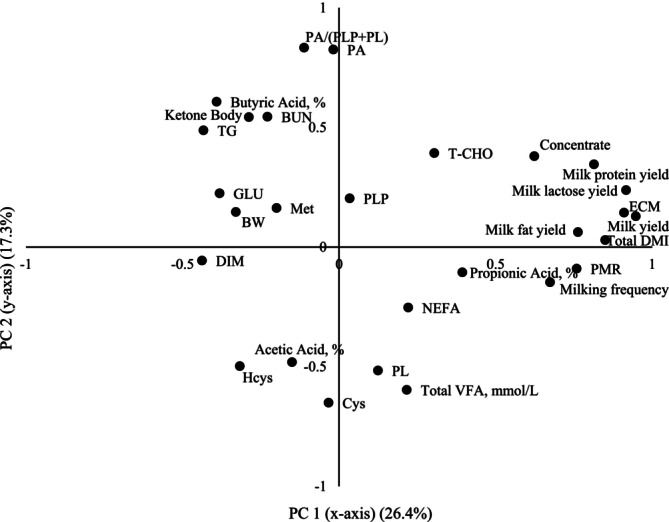
Loading plot of principle component (PC) 1 (*x*‐axis) and PC 2 (*y*‐axis) presenting relationship of plasma vitamin B_6_ vitamers, plasma metabolites, rumen fermentation profile, and milk production parameters with PC1 and PC2, which indicate milk production performance and nutritional condition of cows, respectively. PC1 and PC2 explained 26.4% and 17.3% of variance, respectively.

For PCA, the scree plot indicated an elbow of eigenvalue at PC4 (eigenvalue = 1.9), but PC1 (eigenvalue = 7.4, variance = 26.4%) and PC2 (eigenvalue = 4.9, variance = 17.3%) were retained for further analysis as they captured the possible relevant variance (43.7%). The loading plot of PC1 (*x*‐axis) and PC2 (*y*‐axis) was shown in Figure 1. The results suggest no clear relationship between plasma concentration of vitamin B_6_ (PL, PLP, and PA) with PC1. The PC1 could represent milk production performance because high factor loadings of PC1 were found for milk yield (0.95), ECM yield (0.91), milk lactose yield (0.92), milk protein yield (0.81), and milk fat yield (0.76). In contrast, based on the factor loadings of PC2, plasma PA (0.83), and PA/(PLP + PL) (0.83) had a positive relationship but plasma PL (−0.52) and Cys (−0.65) had a negative relationship with PC2, which could represent variation in nutritional status, metabolic metabolism and rumen fermentation. In addition, based on the loading plot, total ruminal VFA concentration was considered to be closely related to plasma PL. This further supports the relationship of plasma vitamin B_6_ concentration with rumen fermentation. The previous findings reported that dietary factors like digestible NDF content had a negative relationship with apparent ruminal vitamin B_6_ production (Brisson et al. 2022). Furthermore, contrast to PL and PA, plasma PLP has no clear relationship with both PC1 and PC2, which indicates that plasma PLP could be maintained its stable concentration by the conversion from other vitamin B_6_ vitamers under various nutritional and environmental conditions.

In conclusion, the status of vitamin B_6_ in dairy cows milked with AMS appears to meet the demand to support metabolic functions under cold environmental season. Although the vitamin B_6_ status evaluated by plasma PLP concentrations remained stable across seasons, the reduction of vitamin B_6_ turnover under exposure to a hot environment possibly compensates for their demand for metabolic reaction in dairy cows during summer.

In addition, under various feeding and environmental conditions, vitamin B_6_ status may not be directly associated with milk production or feed intake within the range observed in this study. However, variations in rumen fermentation are found to influence the concentrations of plasma vitamin B_6_ vitamers and PA. Assessing plasma concentration of PLP alongside PL, PA, plasma Hcys, and ruminal fermentation profiles, as well as dietary vitamin B_6_ intake, provides a more comprehensive understanding of vitamin B_6_ status in lactating dairy cows.

## Conflicts of Interest

The authors declare no conflicts of interest.
